# Effect of L-Ornithine application on improving drought tolerance in sugar beet plants

**DOI:** 10.1016/j.heliyon.2019.e02631

**Published:** 2019-10-13

**Authors:** Hebat-Allah A. Hussein, B.B. Mekki, Marwa E.Abd El-Sadek, Ezzat Ebd El Lateef

**Affiliations:** a Botany and Microbiology Department, Faculty of Science (Girls Branch), Al Azhar University, Cairo, Egypt; b Biology Department, University College of Nayriya, Hafr Al Batin University, Saudi Arabia; c Field Crops Research Department, National Research Centre, Dokki, Giza, Egypt

**Keywords:** Agricultural science, Biochemistry, Plant biology, Sandy soil, Sugar beet, Drought, Ornithine, Clay soil

## Abstract

The objective of this research was to determine the foliar application of L-ornithine (0.0, 0.30 and 0.60 mM) as a precursor of polyamines, at vegetative stage was on antioxidant defense and growth of drought stressed sugar beet plants grown under clay and sandy soil conditions. Two water irrigation treatments (80% and 40% Field capacity) were carried out on sugar beet plants grown in pots under greenhouse conditions. Water stress resulted in significantly decrement in growth parameters including root diameter, root and shoot weights per plant compared with corresponding control plants. The results showed that drought stress significantly affected most biochemical characteristics of plants. Photosynthetic pigments contents, free amino acids and peroxidase enzyme activity were decreased, while catalase enzyme activity and lipid peroxidation was increased with drought stress. On the other hand, foliar application of L-ornithine effectively alleviated harmful effects caused by drought stress on root length, root and shoot weights of sugar beet plants, especially under sandy soil conditions. The results cleared that ameliorating the negative effects of drought stress through exogenously applied L-ornithine associated with improved photosynthetic pigments, protein profile, lipid peroxidation, antioxidant enzymes; catalase and peroxidase, total soluble sugars and total amino led to increasing drought tolerance of sugar beet plants.

## 1 Introduction

Sugar beet (*Beta vulgaris* var. saccharifera L.) is considered to be the 2^nd^ important sugar crops after sugar cane, producing about 40 % of sugar production annually all over the world. Recently, sugar beet is an important winter crop in the calcareous, saline, alkaline, poor and fertile soils. The great importance of sugar beet crop is due to its ability to be grown in the newly reclaimed areas as an economic crop as well as for its higher production of sugar under these conditions as compared with sugar cane. Most of these areas face some stress problems, i.e. salinity and insufficient of nutrient elements. Water scarcity and drought conditions especially in arid and semiarid places caused by climate change is considered the main factor for affecting on yield and productivity of agriculture crops in many regions around the world (Riccardi et al., 2016). Also, water is essential for vegetative growth for obtaining maximum yield and increasing the crop productivity (Zingaretti et al., 2012; Darwesh et al., 2019).

Modern agriculture to increase water use efficiency and water stress resistance is needed for improving the plant growth performance and crop productivity by using anti-stress compounds. This property can be achieved by tending stabilizing cell structure and/or developing osmotic adjustment. In the Egyptian agricultural strategy, a great attention is being devoted to search for untraditional natural and safe stimulating growth substances to mitigate stress and protect the plant cell from severe damage which have marked influence on plant growth parameters that is reflected to increase plant productivity (El-Aal and EL-Rahman, 2014; Darwesh et al., 2018).

L-ornithine is the precursor of polyamines that are essential in the regulation of plant growth and development (Martin-Tanguy, 2001). Furthermore, it is the intermediate compound in the arginine biosynthesis where the pathway divaricates to the production of compounds, such as proline that serve as osmoprotective substance in plants (Kalamaki et al., 2009; Ali et al., 2016). Polyamines affect DNA, RNA and protein biosynthesis, promote plant growth and development, delay ageing, and improve disease resistance in plants, since polyamines can clean free radicals in plants, they also protect the membrane to some extent from oxidative damage and the most effective polyamines for alleviating drought stress in rice is spermine (Liu et al., 2018).

It is well known the importance of polyamine as plant growth regulators and the positive role under many stresses but they are really, very expensive materials. The high cost of polyamines (spermidine or spermine) led us to search on another alternative source (their precursor that converts into polyamines) with lower cost and more available that allows us obtaining the positive effects of polyamines with lower cost. Therefore, the main objective of the current study is attempt to study the effect of the foliar application of L-ornithine to mitigate the negative effects of drought stress on sugar beet plants sugar beet grown under sandy or clay soil conditions. Moreover, the secondary objective is determination of the soil type (clay and sand) give the best results with the foliar application of L-ornithine on sugar beet plants.

## 2 Materials and methods

### 2.1 Plant material and experimental design

A pot experiment was conducted in the green house of the National Research Centre during the winter season of 2016/17 in clay and sandy soils to determine the effect of foliar L-ornithine application on sugar beet plant grown under water stress conditions. Each experimental set included 6 treatments, which were the combinations between two water irrigation treatments (normal irrigation represented by 80% FC. and water stressed represented by 40% FC and three foliar treatments with L-Ornithine (Orn) at 0, 0.30 and 0.60 mM.

Sugar beet seeds of Halawa variety were sown in earthenware pots No. 50 on November 5th. Each pot contained 30 kg of clay soil obtained from a private farm in Kalubia governorate while the sandy soil was obtained from the Agricultural Research Station of the National Research Centre in Nubaria. Phosphorus fertilizer was added before sowing at a rate of 6.0 g per pot of calcium super phosphate (15.5% P_2_O_5_). Nitrogen fertilizer was applied as two equal portions at a rate of 0.60 g/pot for each in the form of ammonium nitrate (33.5%N) at 30 and 60 days after planting (Elshahawy et al., 2018). Potassium fertilizer was applied as soil application at the rate of 2 g/pot in the form of potassium sulfate (48–52% K_2_O) at 45 days after planting. The experimental design was factorial in three replicates where a factor was soil types, factor B was water regimes and L-ornithine application was the factor C. The first irrigation regime was commenced after 30 days from complete germination and thinning. After 60 days from planting, L-ornithine treatments (0, 0.30 and 0.60 mM) were applied at 5 ml/plant (until both sides of leaves of treated plants were completely wet). Tween-20 at 1% (v/v) as a surfactant was added to l-ornithine solutions and control treatment.

At 75 days after planting a representative sample was taken from each treatment to determine some growth characters; root length, root diameter, root weight and shoot weight per plant and at the same time, the medium leaf was taken from each treatment for determining some chemical analyses.

### 2.2 Photosynthetic pigments

Chlorophyll a, Chlorophyll b and total carotenoids were extracted from 0.1 g of fresh leaves in 10 ml of 85% acetone and measured spectrophotometrically according to Hussein et al. (2019a) and their values were calculated according to the formula of Eida et al. (2018).

### 2.3 Total soluble sugars

Total soluble sugars were determined in ethanol extract of sugar beet leaves by anthrone method according to Cerning and Jutta (1975).

### 2.4 Total free amino acids

Total free amino acids content was estimated according to the method described by Yemm et al. (1955).

### 2.5 Lipid peroxidation

Lipid peroxidation was measured by determining the levels of malonadialdehyde content using the method of Hodges et al. (1999).

### 2.6 Assay of enzymes activities

Enzyme extract was prepared for the assay of different enzymes activities using VEB Carl Zeiss spectrophotometer (Mukherjee and Choudhuri, 1983). Catalase (CAT, EC 1.11.1.6) activity was determined by measuring the decomposition rate of H_2_O_2_ in 60 s with spectrophotometer at 250 nm (Darwesh et al., 2015). Peroxidase (POD, EC 1.11.1.7) activity by measuring the conversion of one micromole of H_2_O_2_ per minute at 25 °C spectrophotometrically within 60 s at 470 nm (Darwesh et al., 2019).

### 2.7 Proteins pattern

Protein extract was carried out in plant leaves, then proteins profiling of samples was performed using SDS-polyacrylamide gel (Laemmli, 1970). At end of electrophoresis, gel was dye in coomassie blue G-250 for 45 min. Then gel fixed in mixture solution of Acetic acid and Ethanol (10%: 40%) overnight on a shaker. After fixing, gel was washed with distilled water (Barakat et al., 2017).

### 2.8 Statistical analyses

The data were statistically analyzed (Sendecor and Cochran, 1980). The least significant differences (LSD) at 5% level of probability were calculated to compare the means of different treatments.

## 3 Results

### 3.1 Plant growth parameters

Plant growth parameters were affected by drought stress and by the foliar application of L-ornithine. In sugar beet plants grown in clay soil (Table 1), water deficient stress significantly decreased (*P* ˂0.05) root fresh weight but significantly increased root length per plant. In sandy soil plants, water stress resulted in increment of root length, decrement in root and shoot weights per plants and no change on root diameter compared to the corresponding control values.

**Table 1:** Effect of water regime and foliar application with L-ornithine on growth parameter of sugar beet plants under clay and sandy soil conditions.

Treatments			Root length (cm)	Root diameter (cm)	Root fresh weight (g)	Shoot fresh weight (g)
Soil type	Water Regime	L-Orn. mM				
Clay	Normal irrigation	0	19.00	5.63	432.50	97.5
		0.30	17.50	6.13	483.75	110.0
		0.60	12.25	5.13	510.00	116.3
	Drought irrigation	0	22.5	4.38	393.75	100.0
		0.30	20.5	5.38	426.25	105.0
		0.60	20.25	5.75	502.5	118.8
Sandy soil	Normal irrigation	0	35.5	8.0	481.25	120.0
		0.30	37.5	6.0	469.0	118.8
		0.60	38.75	6.75	470.0	110.0
	Drought condition	0	36.25	6.25	400.0	107.5
		0.30	41.75	7.50	426.3	111.3
		0.60	40.75	5.50	408.75	100.0
LSD at *P* < 0.05 for drought			2.51	1.69	16.99	3.34
LSD at *P* < 0.05 for L-Ornithine			5.98	1.24	54.24	12.81
LSD at *P* < 0.05 for the interaction			9.0	NS	NS	NS

Under clay soil conditions, foliar application of L-ornithine at high concentration showed positive significant effects on weights of root (510 and 502 g/plant) and shoot (116.30 and 118.8 g/plant) under normal irrigation and drought stress compared to those of control values (432.50, 393.75 g/plant and 97.5, 100 g/plant), respectively. Concerning plants grown in sandy soil, foliar application of L-ornithine, especially at low concentration improved root length and root diameter, root and shoot weight of stressed plants compared to those of untreated stressed plants (Table 1).

### 3.2 Photosynthetic pigments

**Fig. 1:**
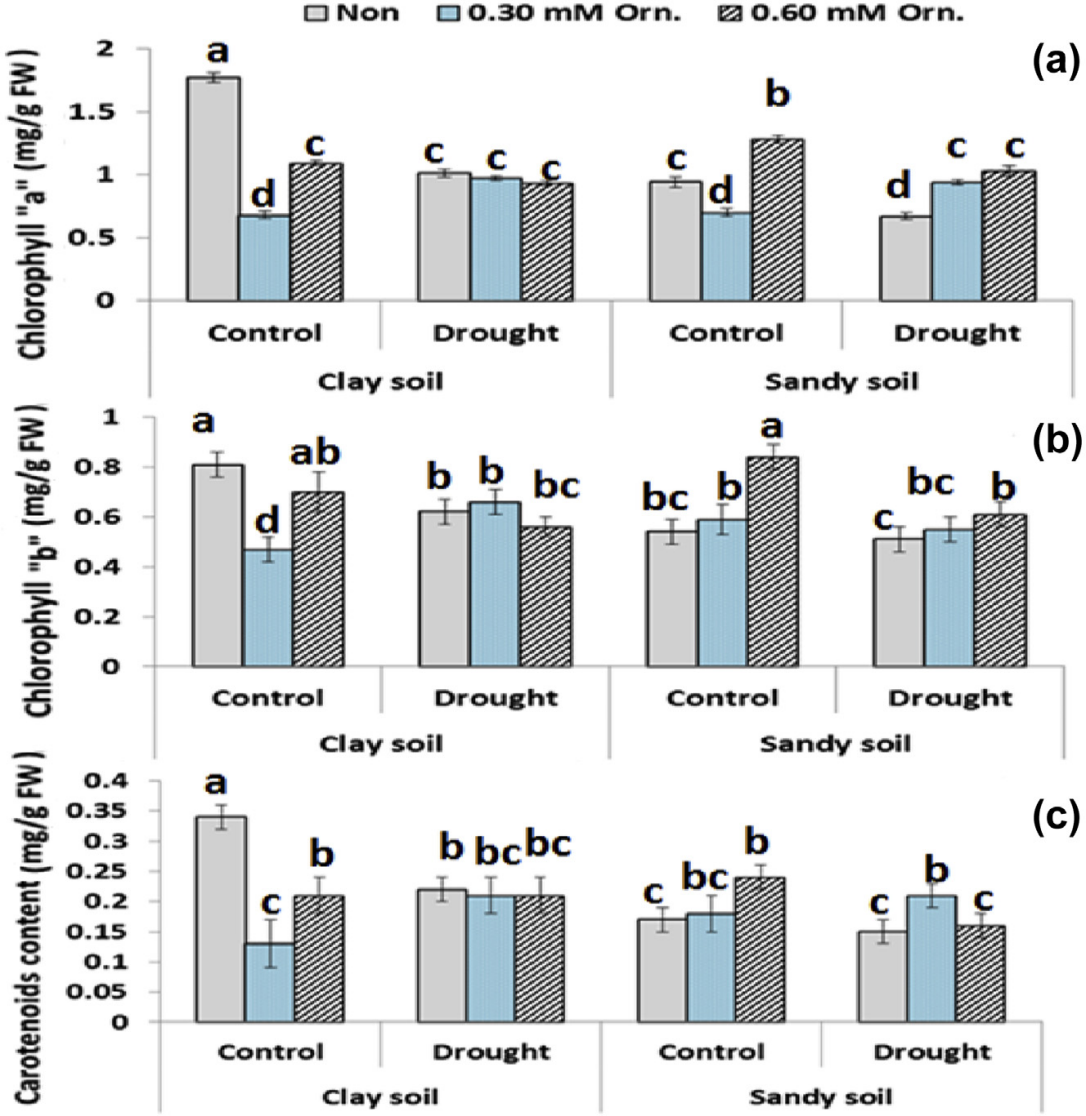
Effect of water regime and foliar application with L-ornithine on chlorophyll “a” (a), chlorophyll "b" (b) and carotenoids (c) content in leaves of sugar beet plants under clay or sandy soil conditions. LSD at *P* < 0.05 for drought 0.20, for treatments 0.25, for interaction 0.30, Vertical bars indicate ±SD.

Data represented in Fig. 1 (a, b, c) showed that drought stress decreased photosynthetic pigments in sugar beet plants grown in sandy soil or clay soil compared to those of the normal irrigated plants. Under sandy soil conditions, high concentration of L-ornithine improved the chlorophylls "a" and "b" in all treated stressed or unstressed plants. In addition, L-ornithine at high concentration significantly increased carotenoids content under normal irrigation, however, low concentration of L-ornithine showed significant increase in carotenoids under drought stress.

### 3.3 Total soluble sugars

**Fig. 2:**
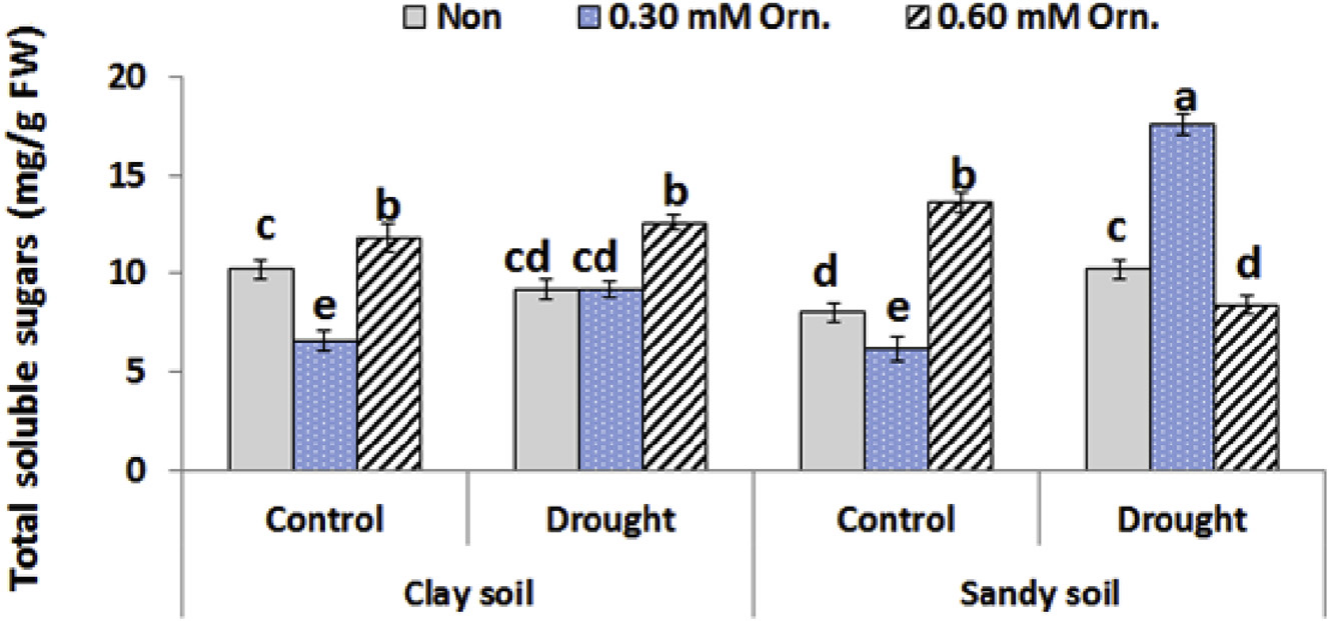
Effect of water regime and foliar application with L-ornithine on total soluble sugars content in leaves of sugar beet plants under clay or sandy soil conditions. LSD at *P* < 0.05 for drought 1.0, for treatments 2.0, for interaction 3.0, Vertical bars indicate ±SD.

The soluble sugar content was significantly decreased (*P* ˂ 0.05) in water deficit plants grown in clay soil. Magnitude was reported in water deficit plants grown in sandy soil compared to the corresponding control values as shown in Fig. (2). Under normal irrigation either in clay or sandy soil conditions, L-ornithine at high concentration resulted in total soluble sugars contents increments in tested plants. Meanwhile, the highest TSS content was achieved in water deficit plants grown in sandy soil and treated with 0.30 mM L-ornithine.

### 3.4 Total free amino acids

**Fig. 3:**
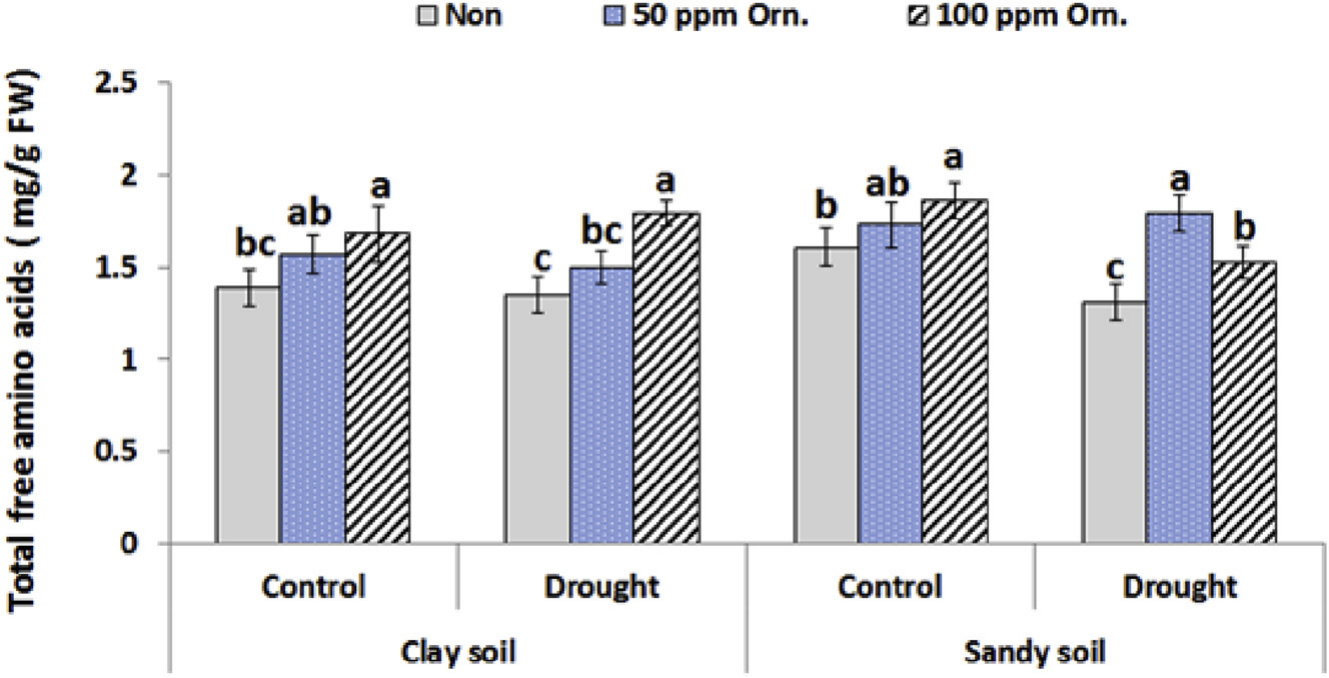
Effect of water regime and foliar application with L-ornithine on total free amino acids content in leaves of sugar beet plants under clay or sandy soil conditions. LSD at *P* < 0.05 for drought 0.10, for treatments 0.15, for interaction 0.25, Vertical bars indicate ±SD.

It is clear that drought stress decreased total free amino acids in plants grown either in clay or sandy soil (Fig. 3). Application of L-ornithine especially at high concentration increased total free amino acids in water deficit and normal irrigated plants grown in clay soil and water deficit plants grown in sandy soil. Therefore, application of L-ornithine especially at low concentration increased total free amino acids in water deficit plants grown in sandy soil as compared to that of the corresponding control plants.

### 3.5 Lipid peroxidation (MDA)

**Fig. 4:**
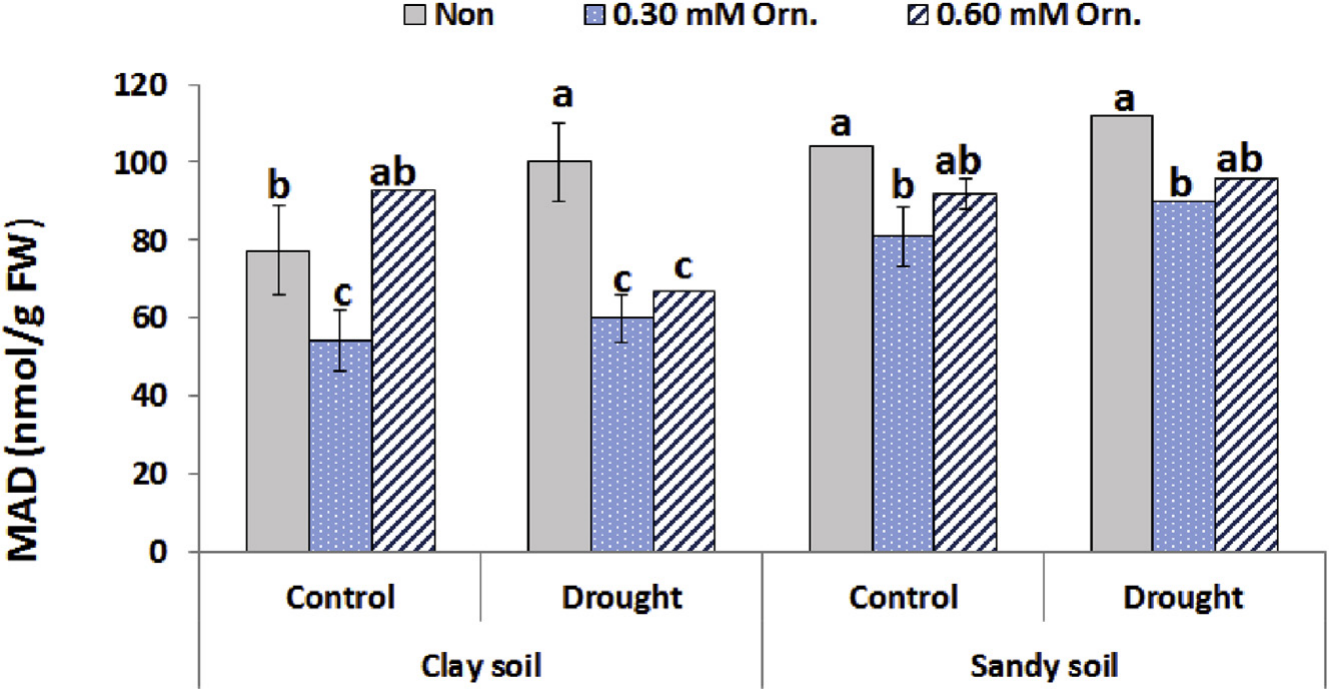
Effect of water regime and foliar application with L-ornithine on lipid peroxidation (MDA) content in leaves of sugar beet plants under clay or sandy soil conditions. LSD at *P* < 0.05 for drought 4.59, treatments 16.0 and for interaction 19.50, Vertical bars indicate ±SD.

The effect of drought on cellular damage indicator (MDA) is shown in Fig. (4). MDA content was increased under drought stress of sugar beet plants either in clay or sandy soil conditions. The level of lipid peroxidation decreased in sugar beet plants with different treatments particularly as the lowest values were recorded in the 0.30 mM Orn treated control and drought-stressed plants (54.3 and 60 nmol/g FW) respectively under clay soil conditions (Fig. 4).

### 3.6 Antioxidant enzymes

**Fig. 5:**
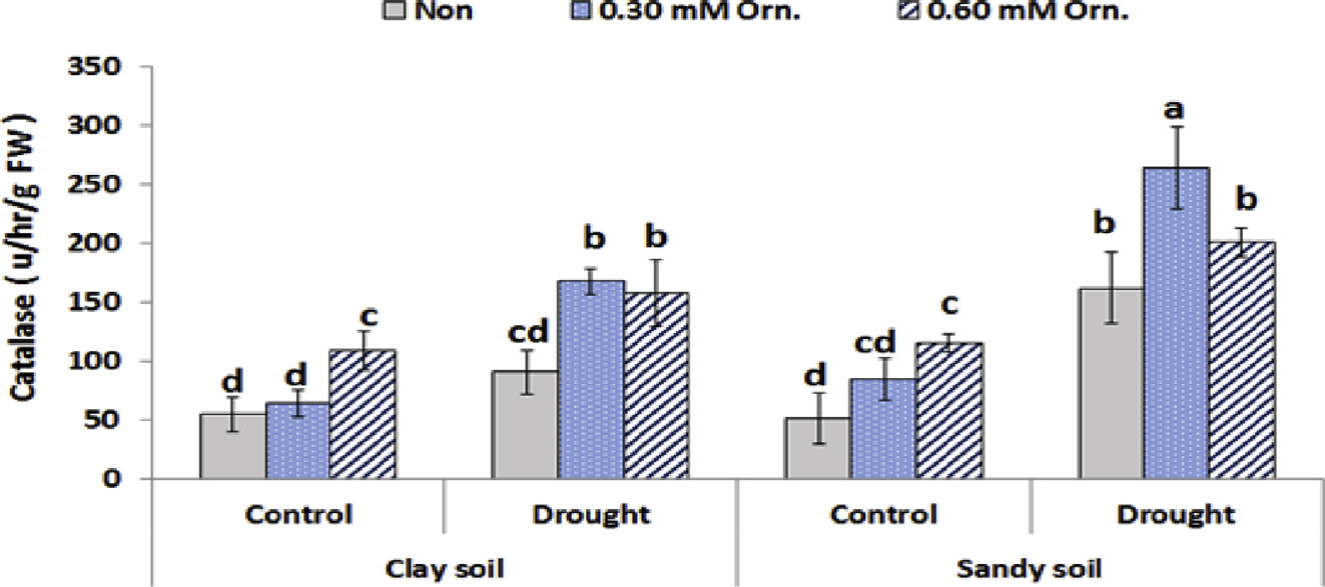
Effect of water regime and foliar application with L-ornithine on catalase enzyme activity in leaves of sugar beet plants under clay or sandy soil conditions. LSD at *P* < 0.05 for drought 9.49, treatments 20.24 and for interaction 25.50, Vertical bars indicate ±SD.

**Fig. 6:**
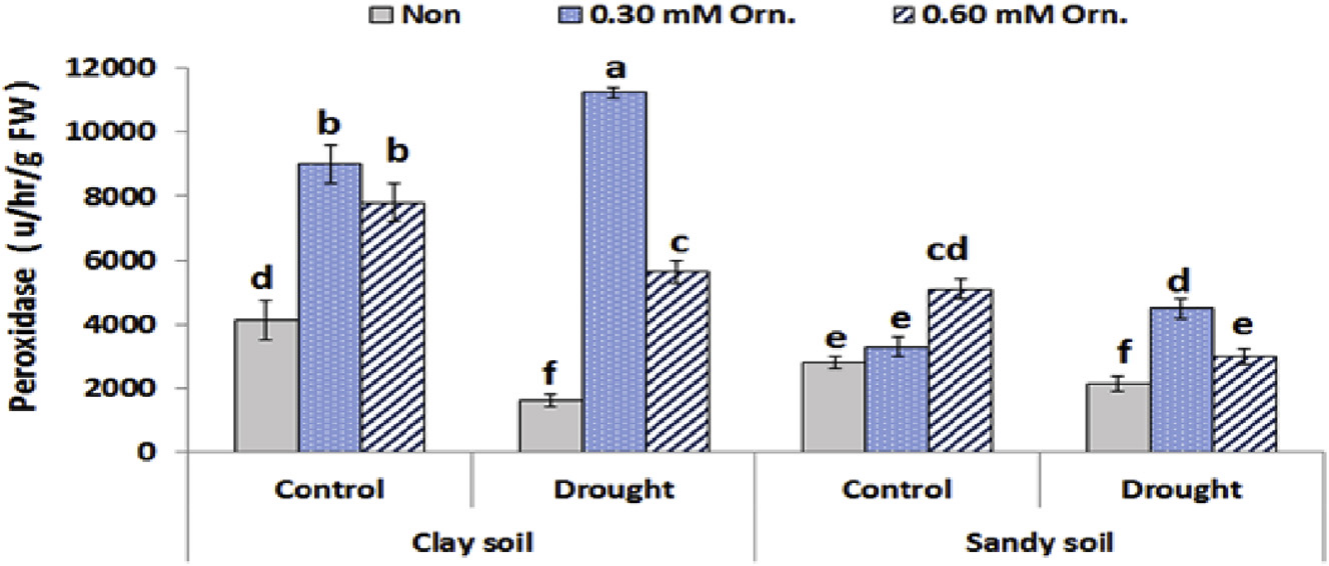
Effect of water regime and foliar application with L-ornithine on peroxidase enzyme activity in leaves of sugar beet plants under clay or sandy soil conditions. LSD at *P* < 0.05 for drought 160, for treatments 182.50 for interaction 353.0, Vertical bars indicate ±SD.

Data given in Figure (5) reveal that catalase enzyme activity was significantly increased (*P* ˂ 0.05) in response to drought stress or L-ornithine application. The highest CAT activity was obtained in water deficit plants treated with 0.30 mM L-ornithine under sandy soil conditions. Concerning peroxidase enzyme (POX) activity, it was significantly decreased (*P* ˂ 0.05) by drought stress under clay or sandy soil conditions. On the other hand, POX activity significantly increased when the plants treated with 0.30 Mm L-ornithine in clay soil as well as the treated water deficit plants grown in sandy soil compared to the corresponding controls (Fig. 6).

### 3.7 Protein pattern

**Fig. 7:**
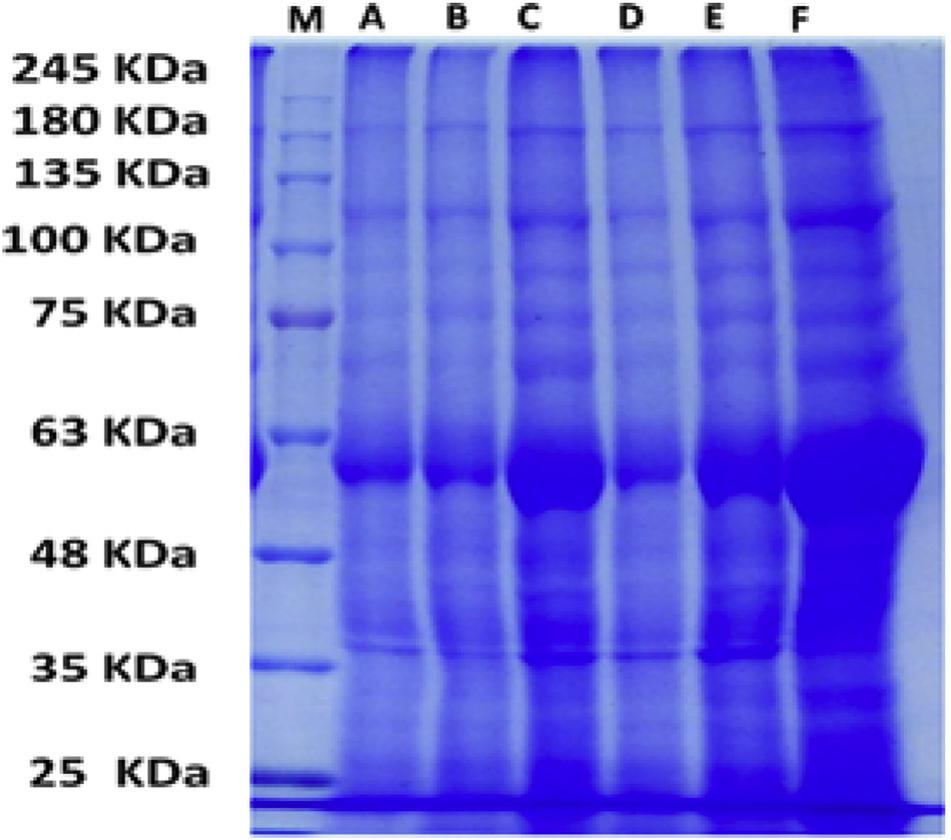
Effect of water regime and foliar application with L-ornithine on protein profile in leaves of sugar beet plants under clay soil conditions. Where A, Control + Normal irrigation; B, Control +0.30 mM L-ornithine; C, Control +0.60 mM L-ornithine; D, Drought control; E, Drought +0.30 mM L-ornithine; F, Drought +0.60 mM L-ornithine.

**Fig. 8:**
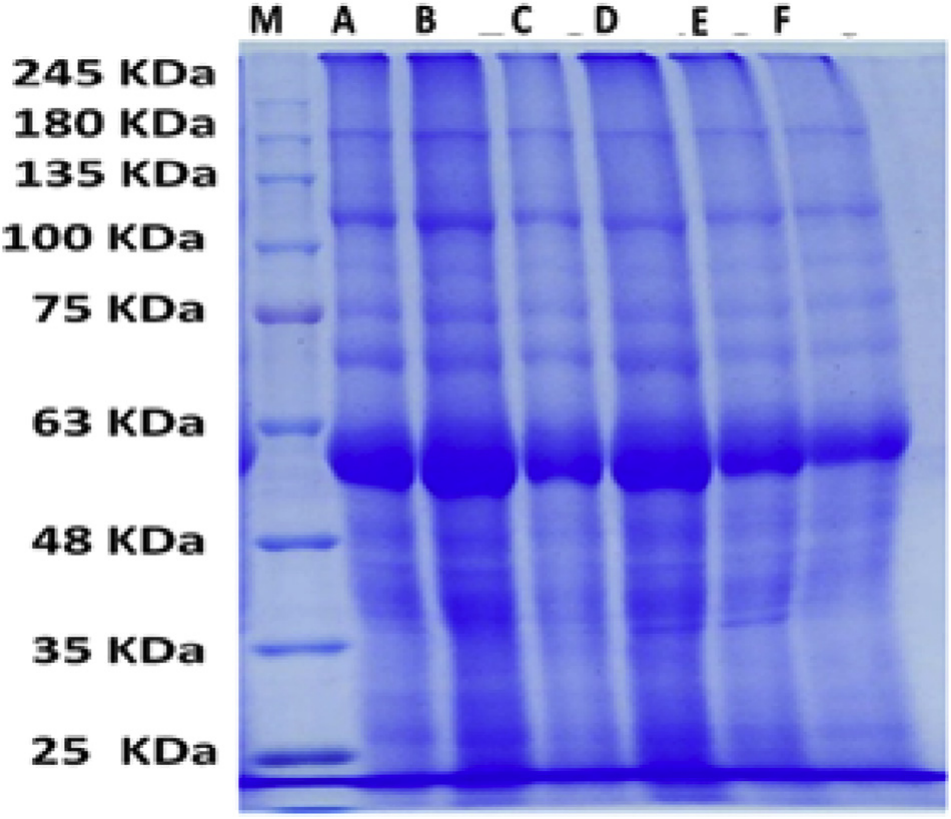
Effect of water regime and foliar application with L-ornithine on protein profile in leaves of sugar beet plants under sandy soil conditions. Where A, Control +60 mM L-ornithine; B, Control + with 30mM L-ornithine; C, Control + Normal irrigation; D, Drought +60mM L-ornithine; E, Drought+ 30 mM L-ornithine; F, Drought control.

Data presented in Table (2) and Figure (7) showed that polypeptide with 165 kDa was present in all L-ornithine treated water deficit plants or normal irrigated sugar beet plants in clay soil. However, polypeptides with 97, 88 & 82 kDa were missed in all tested plants compared to control plants. Furthermore, polypeptide with 46 kDa was induced but polypeptides with 77, 43 and 42 kDa disappeared when all tested plants sprayed with 0.60 mM ornithine in compared to untreated control plants. While under sandy soil conditions (Table 3 and Fig. 8) it is clear that drought stress resulted in disappearance of polypeptides with 140, 100, 83, 75 and 50 kDa compared with control plants. Only, polypeptides with 100, 75 and 50 kDa recovered by Orn treatments. Moreover, polypeptides with 76 and 65 kDa were induced in water deficit plants with or without L-ornithine treatment.

**Table 2:** Effect of water regime and foliar application with L-ornithine on protein profile in leaves of sugar beet plants under clay soil conditions.

No.	M.W	A	B	C	D	E	F
1	250	**-**	**-**	**-**	**-**	**-**	**+**
2	190	**+**	**+**	**+**	**+**	**+**	**+**
3	165	**-**	**+**	**+**	**+**	**+**	**+**
4	135	**-**	**-**	**+**	**-**	**+**	**+**
5	115	**+**	**+**	**+**	**+**	**+**	**+**
6	107	**+**	**+**	**+**	**+**	**+**	**+**
7	97	**+**	**-**	**-**	**-**	**-**	**-**
8	92	**+**	**+**	**+**	**+**	**+**	**+**
9	88	**+**	**-**	**-**	**-**	**-**	**-**
10	82	**+**	**+**	**+**	**-**	**-**	**-**
11	77	**+**	**+**	**-**	**-**	**-**	**-**
12	74	**+**	**+**	**+**	**+**	**+**	**+**
13	70	**-**	**-**	**-**	**-**	**+**	**-**
14	68	**+**	**+**	**+**	**+**	**+**	**+**
15	67	**-**	**+**	**+**	**-**	**-**	**-**
16	66	**-**	**-**	**-**	**+**	**-**	**-**
17	64	**-**	**-**	**-**	**-**	**+**	**+**
18	60	**+**	**+**	**+**	**+**	**+**	**+**
19	54	**+**	**+**	**+**	**+**	**+**	**-**
20	52	**+**	**+**	**+**	**+**	**+**	**-**
21	50	**+**	**+**	**+**	**+**	**+**	**-**
22	48	**+**	**+**	**+**	**+**	**+**	**+**
23	46	**-**	**-**	**+**	**+**	**+**	**+**
24	45	**-**	**-**	**-**	**+**	**-**	**-**
25	44	**+**	**+**	**+**	**+**	**+**	**+**
26	43	**+**	**+**	**-**	**-**	**-**	**-**
27	42	**+**	**+**	**-**	**-**	**-**	**-**
28	40	**+**	**+**	**+**	**+**	**+**	**+**
29	39	**+**	**+**	**+**	**+**	**+**	**+**
30	37	**+**	**+**	**+**	**+**	**+**	**+**
31	34	**+**	**+**	**+**	**+**	**+**	**+**
32	33	**+**	**+**	**+**	**+**	**+**	**+**
33	30	**+**	**+**	**+**	**+**	**+**	**+**
34	28	**+**	**+**	**+**	**+**	**-**	**+**
35	26	**+**	**+**	**+**	**+**	**+**	**+**
36	24	**+**	**+**	**+**	**+**	**+**	**-**

Where A, cont. +normal irrigation; B, cont. +0.30 mM L-ornithine; C, Cont. + 0.60 mM L-ornithine; D, Drought control; E, drought +0.30 mM L-ornithine; F, drought +0.60 mM L-ornithine.

**Table 3:** Effect of water regime and foliar application with L-ornithine on protein profile in leaves of sugar beet plants under sandy soil conditions.

No.	M.W	A	B	C	D	E	F
1	185	**+**	**+**	**+**	**+**	**+**	**+**
2	155	**+**	**+**	**+**	**+**	**+**	**+**
3	140	**-**	**-**	**+**	**-**	**-**	**-**
4	125	**+**	**+**	**+**	**+**	**+**	**+**
5	112	**+**	**+**	**+**	**+**	**+**	**+**
6	100	**+**	**+**	**+**	**+**	**-**	**-**
7	92	**+**	**+**	**+**	**+**	**+**	**+**
8	83	**-**	**-**	**+**	**-**	**-**	**-**
9	76	**-**	**-**	**-**	**+**	**+**	**+**
10	75	**+**	**+**	**+**	**+**	**+**	**-**
11	70	**+**	**-**	**-**	**-**	**-**	**-**
12	67	**+**	**+**	**+**	**+**	**+**	**+**
13	65	**+**	**+**	**-**	**+**	**+**	**+**
14	60	**+**	**+**	**+**	**+**	**+**	**+**
15	57	**-**	**-**	**-**	**-**	**-**	**+**
16	54	**+**	**-**	**+**	**+**	**+**	**+**
17	52	**+**	**-**	**+**	**+**	**+**	**+**
18	51	**-**	**+**	**-**	**-**	**-**	**+**
19	50	**+**	**+**	**+**	**+**	**+**	**-**
20	48	**+**	**+**	**+**	**+**	**+**	**+**
21	44	**+**	**+**	**+**	**+**	**+**	**+**
22	42	**+**	**+**	**+**	**+**	**+**	**+**
23	41	**+**	**+**	**+**	**+**	**+**	**+**
24	39	**+**	**+**	**+**	**+**	**+**	**+**
25	37	**+**	**+**	**+**	**+**	**+**	**+**
26	35	**+**	**+**	**+**	**+**	**+**	**+**
27	34	**+**	**+**	**+**	**+**	**+**	**+**
28	33	**+**	**+**	**+**	**+**	**+**	**+**
29	32	**-**	**-**	**-**	**-**	**-**	**+**
30	30	**+**	**+**	**+**	**+**	**+**	**+**
31	28	**-**	**+**	**-**	**+**	**-**	**-**
32	27	**+**	**+**	**+**	**+**	**+**	**+**
33	25	**+**	**+**	**+**	**+**	**+**	**+**

Where A, Control +60 mM L-ornithine; B, Control + with 30 mM L-ornithine; C, Control + Normal irrigation; D, Drought+ 60mM L-ornithine; E, Drought+ 30Mm L-ornithine; F, Drought conditions.

## 4 Discussion

Drought is considered as the most important environmental stress limiting growth and development. Scarcity of water is a crucial stress factor pointing plant growth through its serious effect on cell enlargements, growth development and elongation (Shao et al., 2008). The negative drought stress effects are due to the imbalance between the production of free radicles and antioxidant defense systems (Hussein et al., 2015). Water deficit is also; alter a variety of biochemical constituents and physiological processes such as photosynthesis, protein synthesis and osmoprotectants accumulation (Mafakheri et al., 2011).

The foliar application of L-ornithine was applied at vegetative stage to sugar beet plants grown under water stress to determine how to alleviate the oxidative damage results from drought stress on sugar beet plant growth. Foliar application of Orn treatments especially at high concentration (0.60 mM) resulted in significant alleviation of drought indications, which seemed as up parameter of general morphological characteristics on plant growth. There is evidence that L-ornithine is the precursor of polyamines that are essential in the regulation of plant growth and development (Martin-Tanguy, 2001). In plants, polyamine has been associated with improvement resistance of drought stress (Kalamaki et al., 2009). Polyamines (PAs) are involved in several physiological processes in plants relating to development in addition to growth and stress responses through a biochemical functions multitude (Quinet et al., 2010). L-ornithine is an important regulator for own biosynthesis as well as biosynthesis and accumulation of glutamine in the cells, and to realize optimal assimilation of carbon and nitrogen leading to increment of biomass production and abiotic stress tolerance in plants (Majumdar et al., 2013).

The composition and content of pigment showed the water stress led to a significant decline in Chl *a* and *b* and carotenoids in sugar beet leaves. The symptoms often clarified as either fast breakdown or slow synthesis of photosynthetic pigments (Smirnoff, 1993). On the other hand, the pigment breakdown was not related to reduction of the efficiency of photochemical parameters (Elsheery and Cao, 2008), but it contributed to increased ratio of carotenoids to total chlorophylls (Liu et al., 2011). Pigment content increased and improved level with foliar application of Orn. To explain this result, we postulate that the Orn metabolic pathway particularly at high concentration may be deviated from polyamines synthesis to alternative pathway which may be lead to over production of certain amino acids, imbalance in the action of several enzymes and/or toxic compound such as H_2_O_2_ lead to thylakoid membrane damage and subsequent decreased the photosynthetic pigments (Youssef et al., 2019). Changes in polyamine biosynthesis and levels are an integral part of the response of plants to stress (Anjum, 2011).

In this respect, the carbohydrates have important role in regulating the plants osmotic pressure and defense substances, on the vegetative stage. The significant effect of drought stress resulted in shifting the level of water soluble sugars. This result may be due to carbohydrates hydrolysis and/or decline in expression of photosynthesis enzymes under water regime stress (Bayramov et al., 2010). In addition, the results showed that total soluble sugars contents decreased in all plants treated with 0.30 mM Orn as compared with the control values. Meanwhile, the opposite results were obtained when 0.60 mM L-ornithine was applied. Lower ornithine level increased the level of polyamine in plant cell (Majumdar et al., 2013) led to alleviate and regulate the harmful effects of drought stress, reflecting in decrement in soluble sugar content in 0.30 mM L-ornithine treated and stressed plants at vegetative stage. On the other hand, high concentration of L-ornithine caused accumulation of soluble sugars, this result might be due to high ornithine level increased arginine and nitric oxide; intermediates in ornithine-polyamine network and act as metabolite signaling molecules and modulate the level of osmoprotective molecules involved in drought tolerance (Majumdar et al., 2013).

The present data revealed slight decrease in free amino acids content under drought stress. The decline in free amino acids under drought stress might be related to the denaturation of enzymes involved in amino acids biosynthesis which leads to the down regulation in protein synthesis of sugar beet plants. Moreover, application of L-ornithine treatments alleviated the oxidative damage consequences of water stress on amino acids with one way or another. Exogenous L-ornithine may be a way to provide plants with a metabolite that can be readily converted to amino acids upon water stress induction (Kalamaki et al., 2009). At the same time, it was found that L-ornithine may play a critical role in regulating not only the metabolism of proline and glutamine (Glu) amino acids but also that of PAs (Elsheery and Cao, 2008). This explanation may be due to L-ornithine levels had a regulatory role in controlling the glutamine to L-ornithine to Arginine and glutamine to L-ornithine to putrescence pathways in plants.

Drought stress is recognized for generating of reactive oxygen species (ROS). It is recognized at the cellular level and scavenged through increasing of anti-oxidative systems (Reddy et al., 2004; Hasanin et al., 2019; Hussein et al., 2019b). The excess of ROS production can cause oxidative stresses in plants by damaging nucleic acids, proteins, photosynthetic pigments and membrane lipids (Yordanov et al., 2000). Antioxidant enzymes are the defense system that constitutes the elimination of reactive oxygen species in plants. The high expression of these enzymes can reduce the damage of reactive oxygen species to plants under stress conditions (Xu et al., 2008).

Under drought stress, the activity of antioxidant enzymes indicated that plant had a higher ability to scavenge free radicals (Wang et al., 2009). Moreover, under drought stress, exogenous L-ornithine helps improve the antioxidant enzyme activity of sugar beet plants, thus enhancing the ability of ROS removal and improving the tolerance of plants. Enhanced antioxidant defense system in l-ornithine treated plants resulted in improving cell membrane stability, as demonstrated by lower lipid peroxidation. L-ornithine might be converted into endogenous polyamines, which may play a dual role in the ROS scavenging process. First, polyamines may play a critical role in drought stress signalling to confer adaptive responses (Pottosin and Shabala, 2014). Moreover, Mustafavi et al. (2016) reported that the polyamines control over the balance between ROS production and scavenging may “shape” H_2_O_2_ signal. In addition, polyamines may form complexes with CAT that are more efficient than the isolated enzyme. A higher peroxidase activity could be explained by a possible role of polyamine–hydroxycinnamic acid conjugates as substrates for this enzyme (Sang et al., 2016).

The appearance of 2 differentially proteins as well as the reappearance of 2 other proteins which disappeared by water stress in response to foliar application of L-ornithine might be predict that there are 4 stress responsive genes related to L-ornithine action in adaptation to drought stress in sandy soil (Barakat et al., 2015). On the other hand, the disappearance of 3 differentially protein with molecular weight 97, 88 and 82 kDa in response to both L-ornithine concentrations and 3 other polypeptides (77, 43 and 42 kDa) only at high concentration of L-ornithine under with or without drought stressed in clay soil. These results mean that high concentration of L-ornithine may highly affect down regulation of 6 genes. Generally, changes in protein expression in sugar beet plants exposed to drought stress and/or treated with L-ornithine may had a potential role in vital physiological processes such as redox regulation or signal transduction via modulating polypeptides responsible for oxidative stress.

## 5 Conclusion

The current study was devoted to highlight results for foliar application of L-ornithine in sugar beet plants grown in pots under water stress conditions. The results showed that foliar application of L-ornithine improved drought tolerance in sugar beet plants by regulation osmotic mechanism such as accumulation soluble sugars and total free amino acids, maintaining the stability of membranes by lowering lipid peroxidation and increasing some antioxidant enzymes and synthesis of new polypeptides. These results are evident for the sugar beet plants grown either in clay or sandy soils. Although, L-ornithine is an expensive amino acid as effector for alleviation of drought stress, it is considered as an important natural compound from economical and scientific views and it may be help plant physiologists to get more knowledge on the mode of action of it in stressed plants. So, the authors recommended with more future studies on the influence of L- ornithine on water relation parameters and proteomic analysis.

## Declarations

### Author contribution statement

H-A A. Hussein, B.B. Mekki, M. E. Abd El-Sadek, Ezzat Ebd El Lateef: Conceived and designed the experiments; Performed the experiments; Analyzed and interpreted the data; Contributed reagents, materials, analysis tools or data; Wrote the paper.

### Funding statement

This work was supported by NRC through the project “Improvement of yield and quality traits of sugar beet using some agricultural treatments in Nubaria”.

### Competing interest statement

The authors declare no conflict of interest.

### Additional information

No additional information is available for this paper.
